# Supplementary figures and images for: The Promyelocytic Leukemia Protein facilitates human herpesvirus 6B chromosomal integration, immediate-early 1 protein multiSUMOylation and its localization at telomeres
Source: PLoS Pathog. 2020 Jul 13;16(7):e1008683. doi: 10.1371/journal.ppat.1008683 (PMC7394443; doi:10.1371/journal.ppat.1008683)

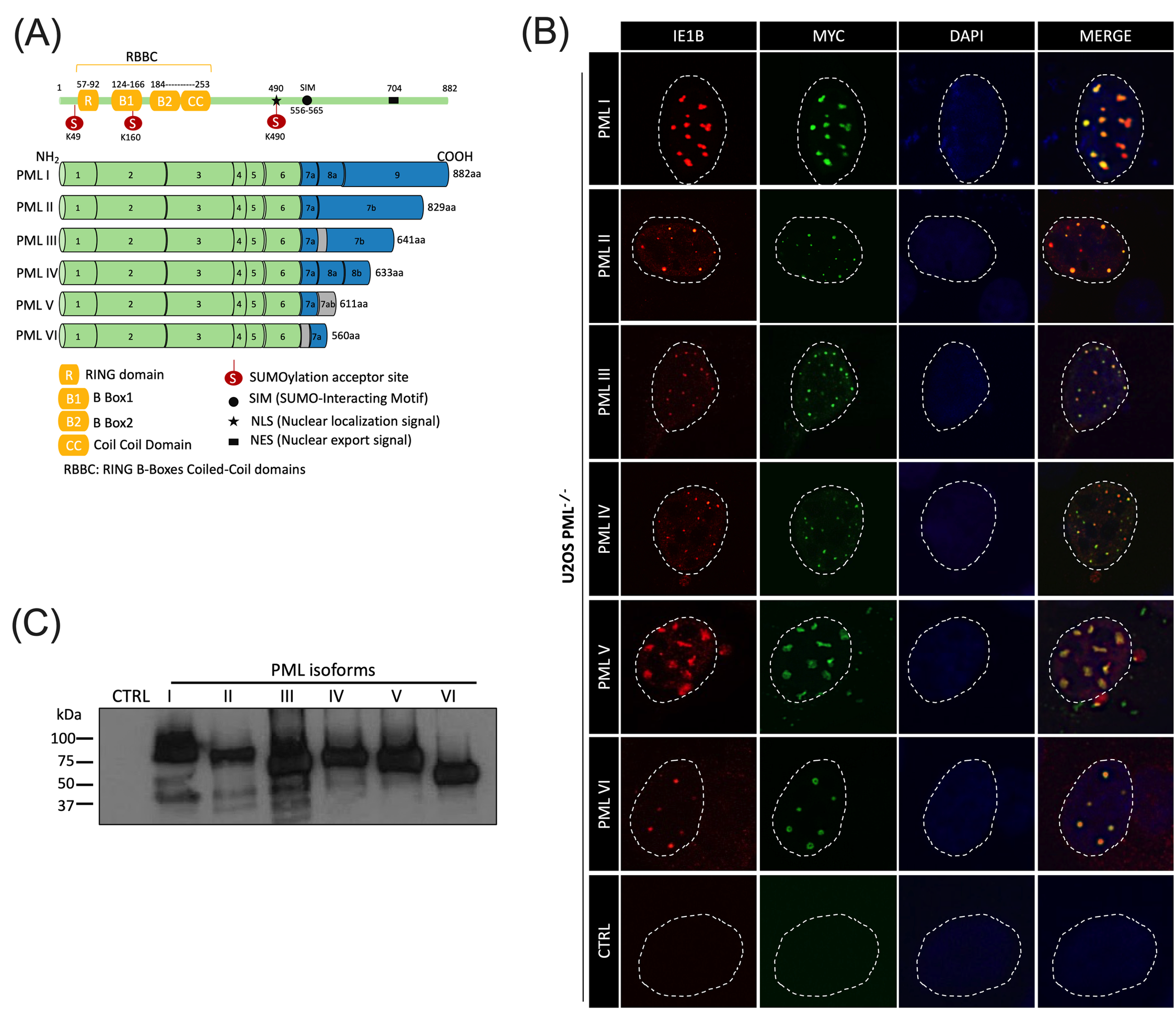

Supplement: S1 Fig — (A) Illustration of the main features of the PML protein and its six nuclear isoforms after alternative splicing. The green and blue colors represent exons and the grey color represents introns. (B) U2OS PML-/- were co-transfected using pcDNA4TO-IE1B along with vectors expressing the various Myc-tagged PML isoforms (I to VI). 48 hours post-transfection, cells were analyzed by IF using anti-Myc ALEXA-488-labeled (green) and anti-IE1 ALEXA-568-labeled (red) antibodies. (C) HEK293T were transfected with Myc-tagged PML isoforms expression vectors and analyzed by western blot using anti-Myc antibody. (TIF) [file ppat.1008683.s001.tif]

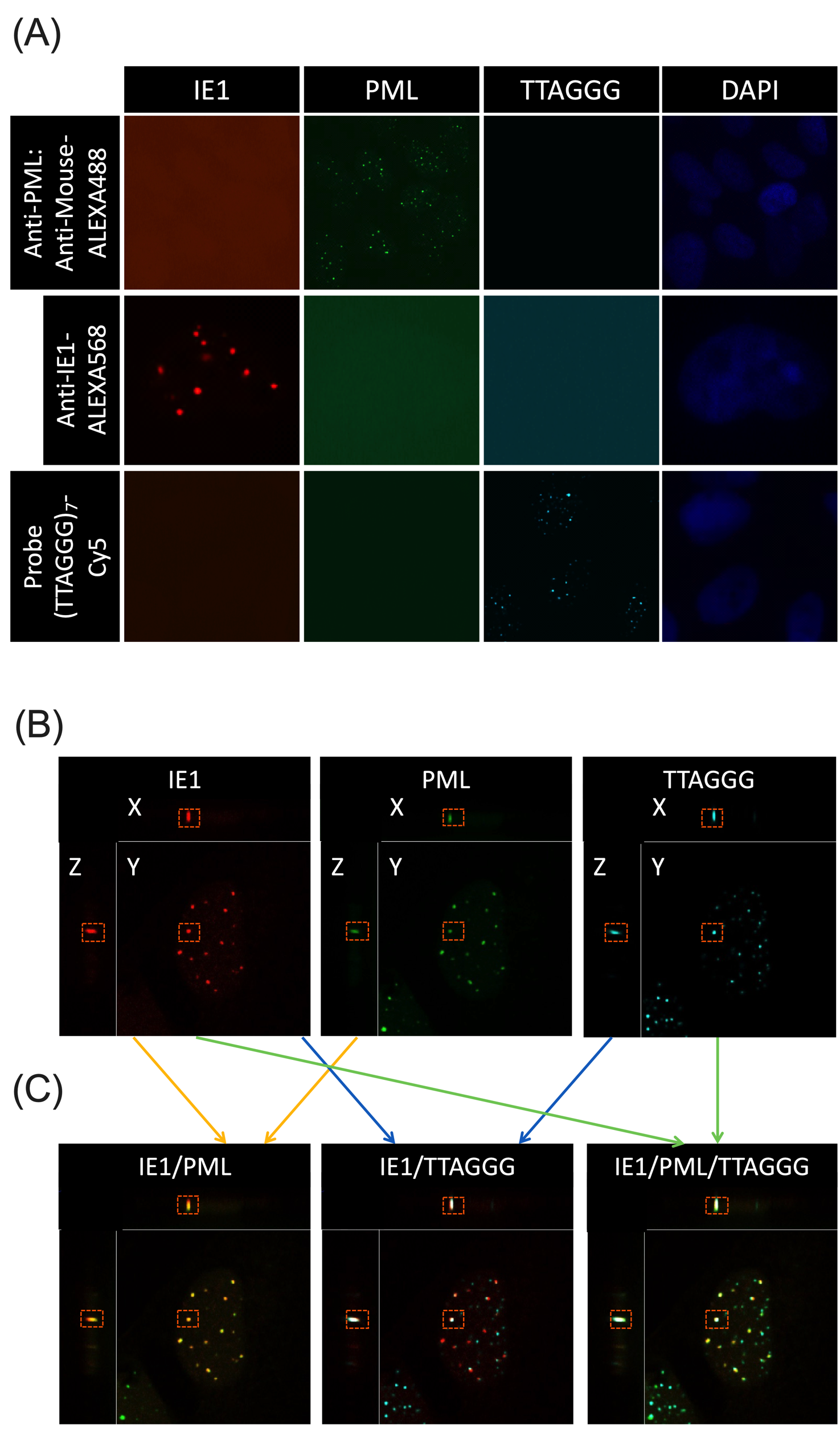

Supplement: S2 Fig — (A) Controls for antibody specificity and fluorescence cross-leakage. U2OS pcDNA4/TO-IE1B transfected cells were labeled separately for anti-PML (green), anti-IE1 (red) and telomeric probe (aqua). Acquisitions were made for each sample to determine whether leakage of fluorophores occurred. All three filters were specific and did not allow fluorescence leakage. (B) Images representing fluorescence of each target protein/DNA in a X, Y, Z manner (3D) (C) Images representing colocalization of IE1 with PML, at telomeres in 3D (orange square). Combination for each channel can be observed. Triple colocalization gives white foci. (TIF) [file ppat.1008683.s002.tif]

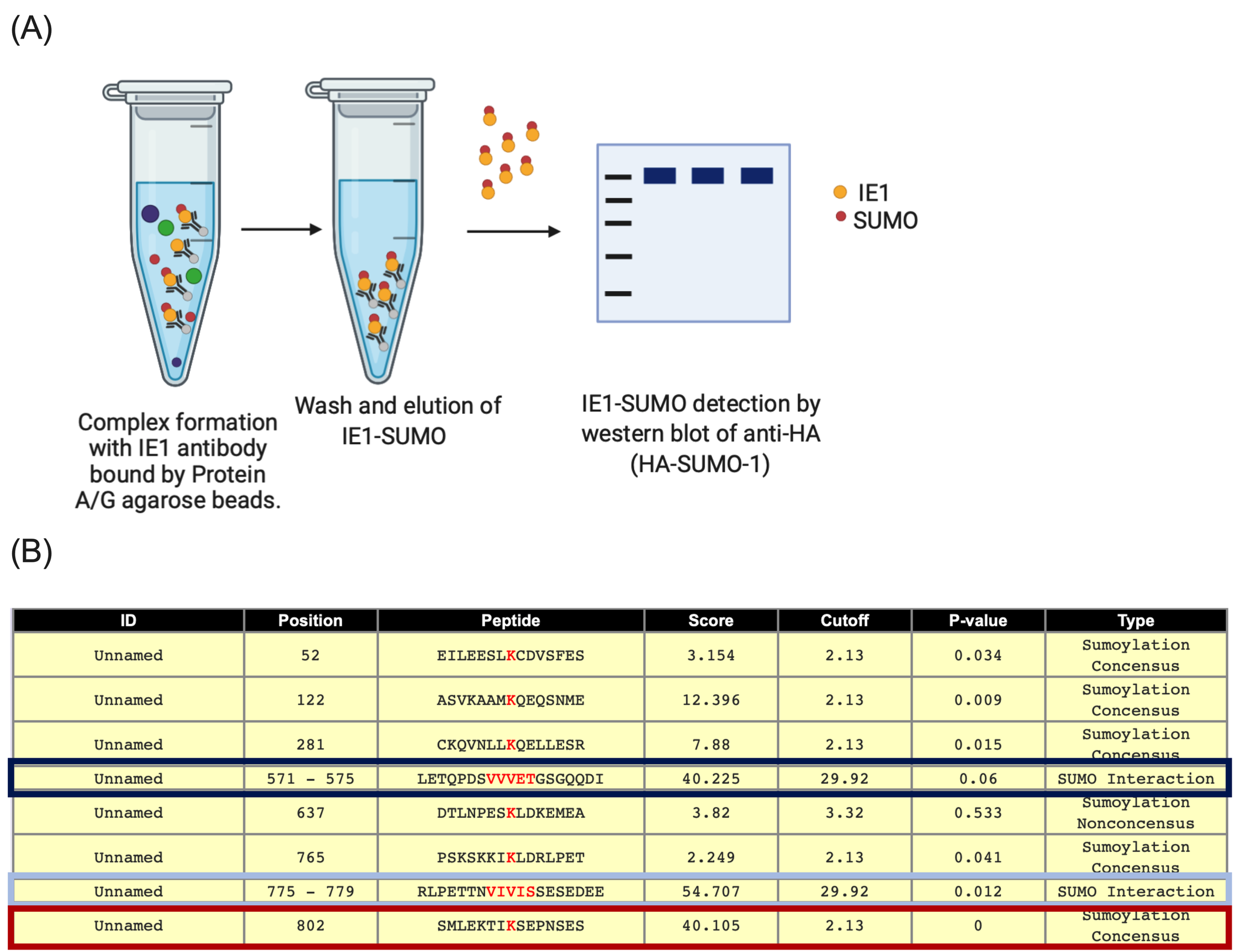

Supplement: S3 Fig — (A) SUMOylation assay protocol in brief. Total cellular lysate is first incubated with IE1 antibody for 1 hour after which Protein A/G agarose beads are added and incubated overnight. After several washes, samples are boiled at 100°C in 2X laemmli sample buffer for 5 minutes. Samples are separated by a 6% SDS-PAGE and SUMOylated IE1 detected using an anti-HA antibody (for HA-SUMO-1 detection on IE1). (B) The amino acid sequence of IE1 was screened (Q77PU6 from UniProt) with the SUMO and SIM site predictor GPS-SUMO (http://sumosp.biocuckoo.org/online.php). Potential SIM sites are indicated by the rectangles based on the calculated p-values. (TIF) [file ppat.1008683.s003.tif]

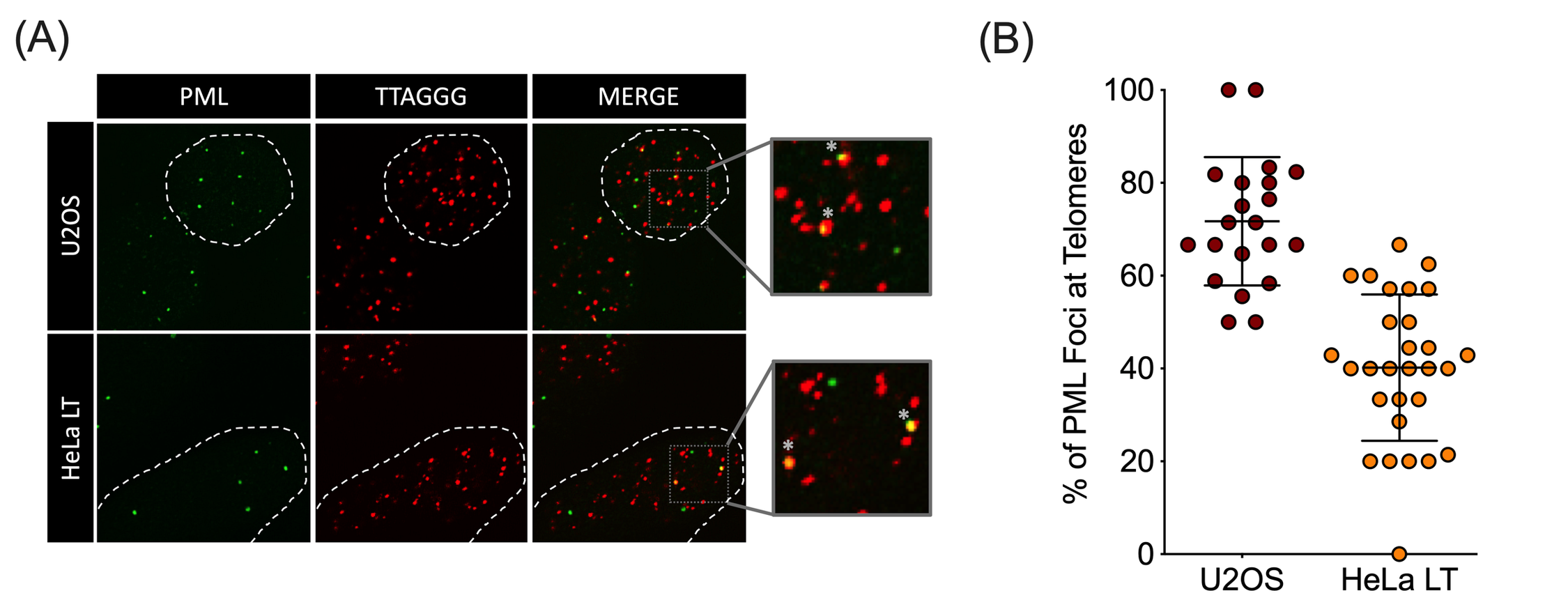

Supplement: S4 Fig — (A) U2OS cells (ALT+) and HeLa LT cells (telomerase+) were grown on coverslips and fixed with 2% paraformaldehyde at sub confluence. Cells were analyzed by IF-FISH. The PML protein was detected using an anti-PML with an anti-mouse-ALEXA-488 (green) antibodies and telomeres were detected using a Cy3-labeled telomeric probe (red). (B) Graph representing mean±sd of the percentage of PML foci localizing at telomeres in U2OS (N = 20) and HeLa LT (N = 40) nuclei. 71.7%±3.01 of PML colocalize at telomeres in U2OS cells and 40.18%±2.92 in HeLa LT cells. (TIF) [file ppat.1008683.s004.tif]

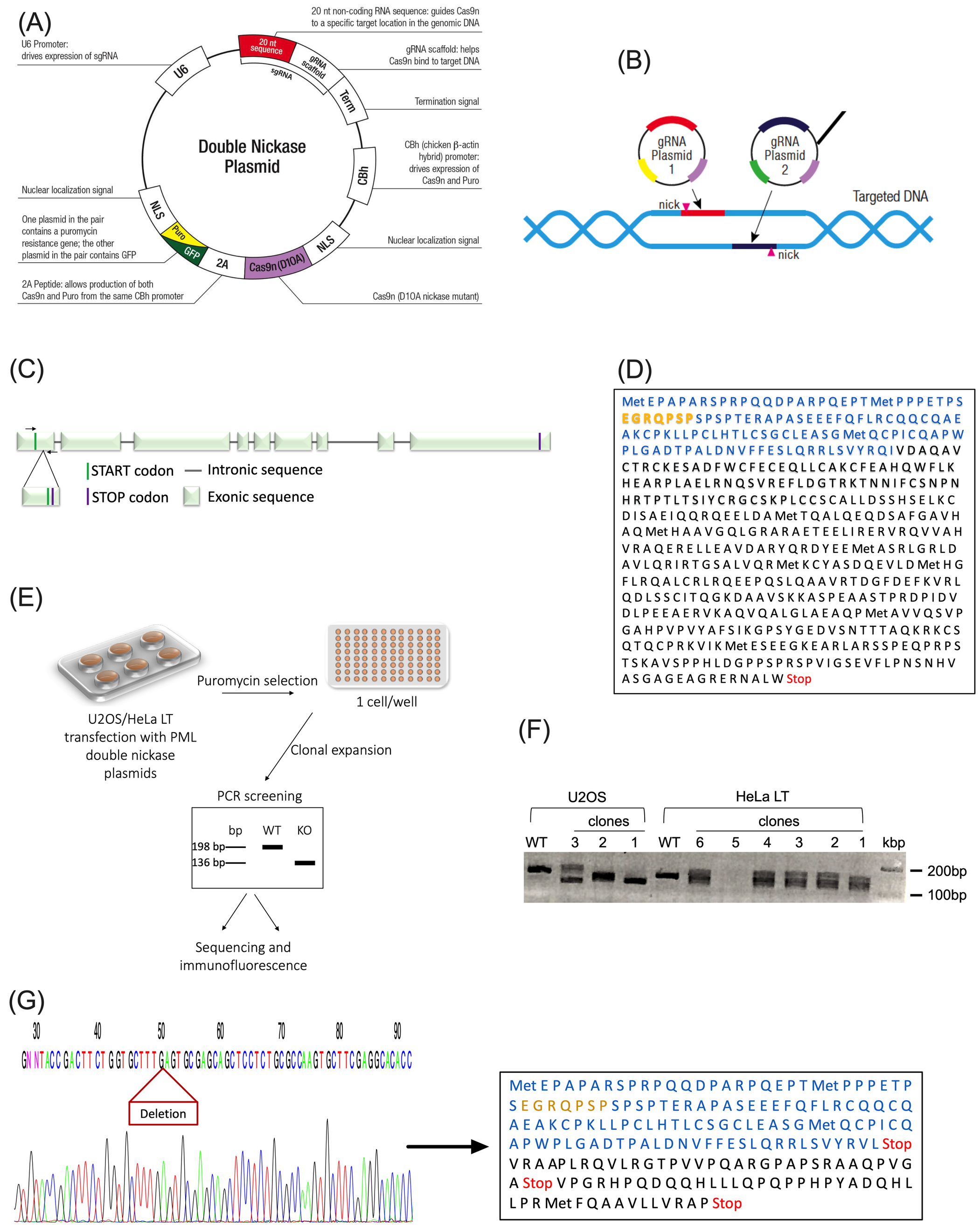

Supplement: S5 Fig — (A) Double nickase plasmid backbone. One plasmid is for a first guideRNA in the leading strand of exon 1 of PML, the expression of a Cas9n(D10A) and the puromycin resistance gene. The second plasmid has a guideRNA in the lagging strand overlapping with the first guideRNA, the expression of a Cas9n (D10A) and GFP. (B) Scheme of the double nickase cuts. (C) Schematic representation of the PMLI gene indicating where in exon 1 the deletion is introduced. Black arrows represent the primers used for PCR amplification. (D) PML gene translation before deletion. Sequence in blue represents the length of the protein after deletion of a part of a sequence in exon 1. Truncated protein is from 1–222 amino acids. Sequence in yellow represents the antigen recognition sequence (aa 31–57) by the monoclonal mouse anti-PML antibody used and purchased from Santa Cruz Biotechnology Inc (SC-966). (E) Experimental procedure used for the generation of PML KO cells. Plasmids expressing Cas-9 and the guide RNAs targeting exon 1 of the PML gene were transfected in U2OS and HeLa LT cells. 48 hours post-transfection, cells went under puromycin selection for a week. Selected cells were then plated into single cell per well in 96-wells plate to do a single cell cloning assay. Wells were screened every week for the presence of a unique colony. Once single cell clones were amplified, they were next transferred into more voluminous wells to amplify the clones and screened. (F) PCR amplifications of WT U2OS and HeLa LT cells and their clones with primers designed as in S5C. When mutated, the PML amplification band is at 136bp instead of 198bp, as observed for U2OS clones. WT and mutant bands were extracted and sequenced. For HeLa LT cells, because this cell line contains more than one chromosome 15 (chromosomal location of PML), these clones had more than one amplification band and were therefore screened by PCR and IF. We have selected clone 1 and 2 for each of the cell line for further experiments. [file ppat.1008683.s005.tif]

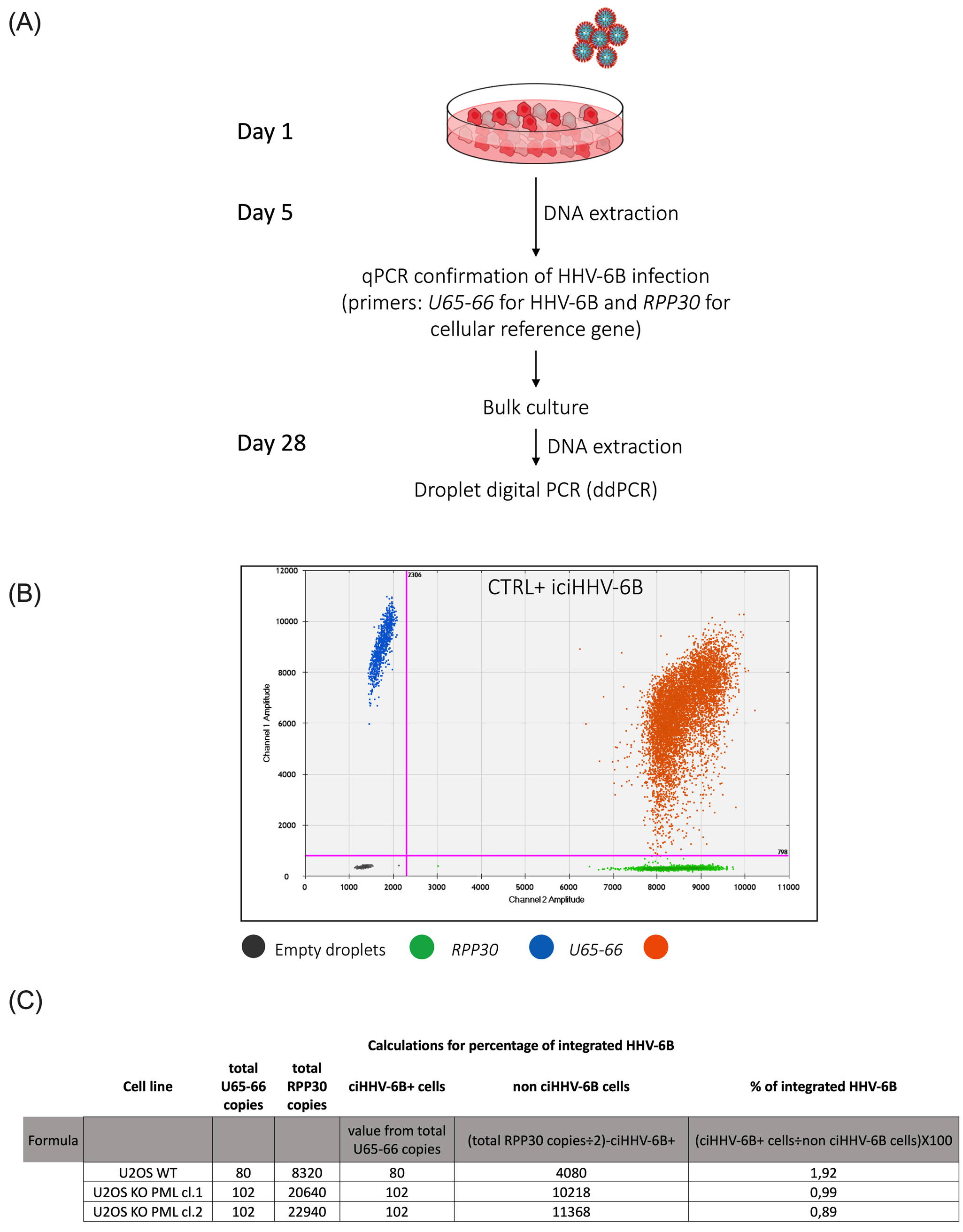

Supplement: S6 Fig — Scheme representing the integration assay as previously described by Gravel et al. (36). Briefly, cells were infected at a MOI of 1 for 24 hours and kept in culture for 4 weeks. At day 5, cells were transferred to a bigger culture vessel and a portion of each condition were screen by qPCR to assure HHV-6B presence. HHV-6B copies were detected with U65-66 primers and normalized with the cellular reference gene (RPP30). (B) Scheme showing the results of an inherited chromosomally integrated HHV-6B sample estimated integration rate by ddPCR. Briefly, cell’s DNA together with a Taq polymerase and primers with their specific fluorescent probe, for instance, are divided into 20 000 droplets of oil. These droplets are then PCR amplified for both of the target genes (U65-66 and RPP30). After the amplification, each droplet is quantified for their number of copies for each gene. (C) Example of calculations to determine the integration rate of HHV-6B. For statistical purposes, the total number of cells with integrated HHV-6B and cells without integration were used. The number of cells is estimated using the RPP30 gene, present at 2 copies/cell. A Fisher’s exact test is done with these values. (TIF) [file ppat.1008683.s006.tif]

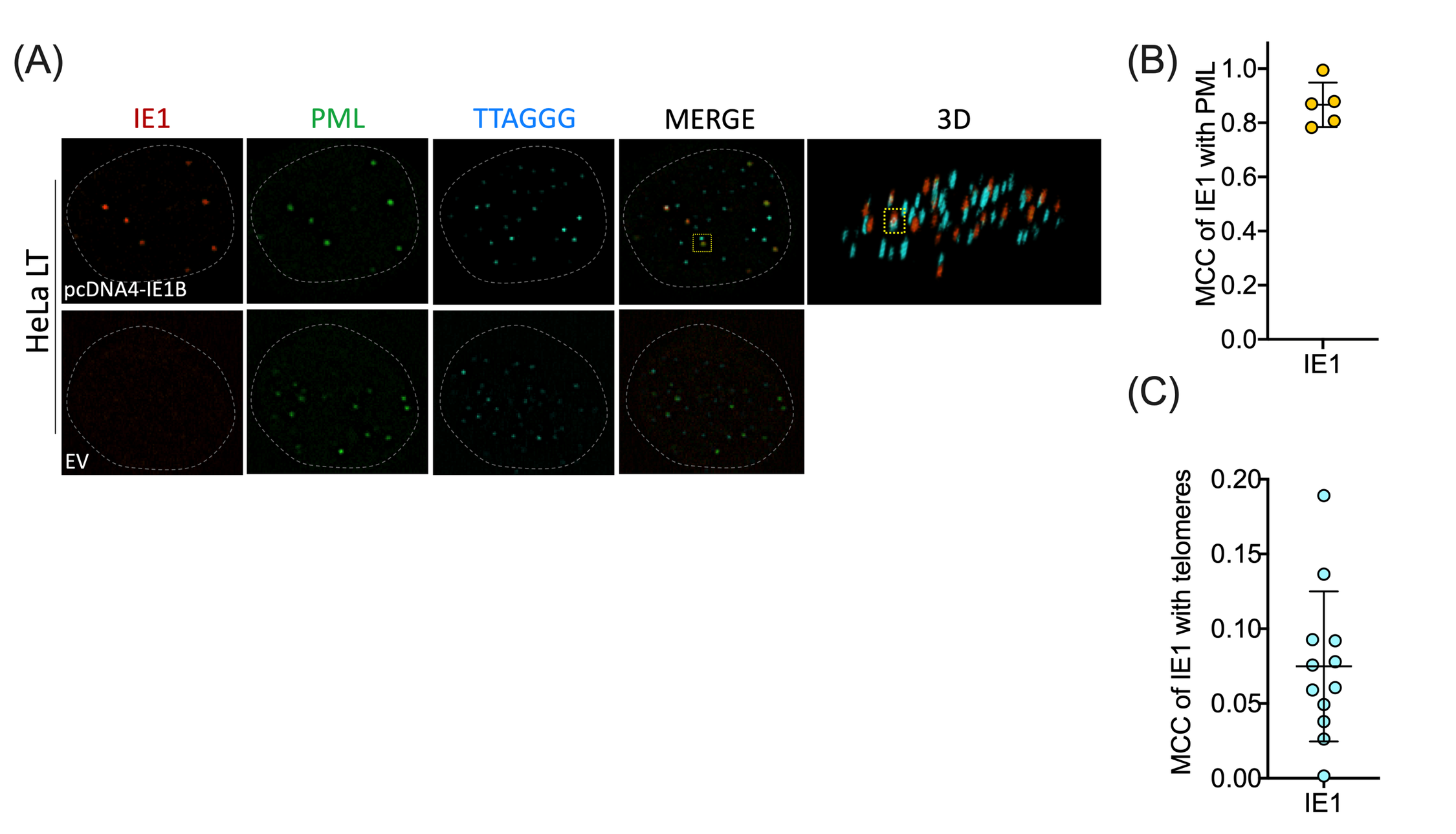

Supplement: S7 Fig — (A) IF-FISH of transfected HeLa LT with pcDNA4/TO-IE1B. 48h post-transfection cells were fixed and labelled for IE1 (red), PML (green) and telomeres (aqua). (B) Graph representing the level of colocalization of IE1 with PML. (C) Graph representing the level of IE1 that localizes at telomeres. (TIF) [file ppat.1008683.s007.tif]
