# Supplementary material for: The Promyelocytic Leukemia Protein facilitates human herpesvirus 6B chromosomal integration, immediate-early 1 protein multiSUMOylation and its localization at telomeres
Source: PLoS Pathog. 2020 Jul 13;16(7):e1008683. doi: 10.1371/journal.ppat.1008683 (PMC7394443; doi:10.1371/journal.ppat.1008683)
Supplement: Attachment — Submitted filename: Collin et al. Rebbutal letter.docx [file ppat.1008683.s008.docx]

Dear reviewers,

Thank you for taking the time to analyze our manuscript and making constructive suggestions. As a result, we have conducted many new experiments that strengthen the manuscript. We provide you with a point by point response to the major issues initially raised.

Key experiments for acceptance

Reviewer #1

1. Nearly the entire paper depends on IFA and findings of co-localization. However, the basis for determining co-localization are not described. All of the foci are different shapes and sizes, thus the degree of overlap is quite varied. Was imaging software applied to measure pixel volume to set a cutoff for a bona fide signal? Was an algorithm applied to determine if two and even three colors had real overlap?

- Before any colocalization quantification was done, we first determined that the antibodies used for colocalization were specific and that there was no emission bleed through between the dyes and filters selection. You can now find the images in figure S1A. Contrast intensity was boosted to demonstrate that there is no bleed through. Moreover, a deconvolution was applied to images to correct if there were any aberrations before analysis.
- Minimum detection exposure time was always used for each dye to avoid overexposure.
- You can find in the method section, further explanations on how the colocalization was quantified. Briefly, we used ImageJ colocalization plugin to calculate colocalization. From high-quality acquisitions, the channels were split (telomeres, PML, IE1 and DAPI) and colocalization could only be analyzed between two channels. For instance, after reduction of the background noises to eliminate false colocalization signal, IE1 protein (channel 2) was quantified for its colocalization with telomeres signal (channel 1) throughout the whole stack (each layers of the nuclei that was acquired). A Costes auto threshold was applied to avoid manual subjectivity. Colocalization is quantified into Manders coefficient (coefficient of colocalization), it gives the proportion of each dye A that colocalize with dye B and vice versa. Indicated values are between 0 and 1 where 1 stands for perfect colocalization, which can be stated that it is 100% colocalization.
- Different forms of foci can be observed because they can be found in every layer of the nucleus, thus, the images shown are only a 1D representation of the nucleus, the smaller foci are further in the density of the nucleus. On the other hand, they are all taken into consideration since the colocalization analysis is carried throughout the the nuclei.
- Because U2OS have heterogenicity in telomere length and in the number of telomeres, this explains why the signal can be stronger or have a different shape.

2. Even where there is co-localization, generally only a fraction colocalizes, for instance ~20% of IE1 colocalizes with telomeres in the presence or absence of the virus. Does the presence of the viral genome influence IE1 and PML co-localization or triple IE1, PML, and telomere localization?

- We do not observe a significant difference between IE1 at telomeres during infection of transfection (see figures 2 and 3)

3. Figure 5- the authors try to connect ALT with PML-NB localization at the telomeres. This is a weak correlation since they are also comparing two different cell types. U20S cells seem to have more overall levels of PML-NBs compared to HeLa.

- This figure is now in a supplementary data as included to show that PML-NB at telomeres could be observed in both cell lines used. We also wanted to confirm published data regarding this, with our own cell lines^1^. It is normal that more PML-NBs are at telomeres in U2OS as this is a hallmark of ALT cells.^2,3^,

4. In the absence of PML, there is less integrated HHV-6 after a month-long infection. This is a very slight decrease with a high degree of variability, as might be expected with this difficult assay. Can the authors further verify a decrease in integration/latent viral load by examining for a decrease in lytic gene expression upon reactivation stimuli (IFA for lytic antigens)? The authors should re-introduce PML to the KO cells to desmonstrate a rescue/restoration of the integration back to control levels.

- We are not certain to fully understand the reviewer’s comment about measuring lytic gene expression after reactivation. Once integrated, it is difficult to reactivate the virus or induce viral gene expression. We have done many such experiments, summarized in Gravel et al. ^4^ The majority of cells containing integrated virus do not express viral genes even when stimulated. This appears to be clone dependent and therefore not a reliable way to assess integration/latency or reactivation.
- Thank you for pointing the restoration of PML into the KO cells. In this new version we present the data following restoration of PML in PML KO cells and its effect on HHV-6B integration (Figure 2G).

5. Lines 374-376: ‘Of note, integration rates are higher in U2OS presumably because of higher constitutive DDR repair in the cells, supporting the hypothesis that DNA repair mechanisms are involved in the HHV-6B integration process (71).’  Presumptions do not support hypotheses. In addition, reference # 71 does not have data regarding HHV-6B.

- Corrected

6. The authors previously demonstrated that IE1 is SUMOylated, but do not demonstrate that SUMO plays a role in the association with PML-NB or if SUMOylation is important for localization to telomeres. In other words perhaps the interaction of IE1 with ‘PML’ is true and IE1 localization to telomeres is also ‘true’, but the role of PML1 or IE1 in each other's localization to telomeres is not demonstrated. The PML-IE1 localization to telomeres has no causal link to the decrease in the efficiency of integration in the absence of PML. Overall the reasoning is shaky and does not demonstrate a direct role for PML.

- We agree with the reviewer and conducted several new experiments. The results are presented in figure 4 and figure 5.
- We know that PML is a SUMO E3 ligase^5^. We now report that PML enhances IE1’s SUMOylation, likely behaving as a SUMO ligase for HHV-6B IE1.
- Furthermore, we report, hyper SUMOylation of IE1 when PML is expressed.
- IE1 contains a SUMO acceptor sites and putative SUMO-Interacting motif (SIM) consensus sequences. We generated mutants of SUMO and SIM sites and report that the VIV SIM site is important for SUMOylation of IE1.
- By adding PML, hyperSUMOylation could be observed for WT IE1, but was lost in the VIV SIM mutants. Therefore, we showed how PML acts on IE1, by hyperSUMOylating IE1 through the VIV site.
- Moreover, we show that the VIV SIM site was required for the formation of bigger IE1/PML foci and that both the VIV SIM and the SUMO acceptor site (K802) are essential for IE1 to adopt its punctate nuclear distribution.

7. Is there any evidence for an increased DNA damage response/repair factors that is linked to loss of PML? Was there any indication of differential DNA damage responses in the PML KO cells in the context of infection?

- In non-neoplastic cells, there is a link between the loss of PML and DNA damage response (DDR). ^1^ However, it seems that immortalized cell lines do not have a difference in DNA damage response weather PML is present or lost.
- We did not look if there was more DDR during infection in WT vs KO cell lines. However, we look at 53BP1 in uninfected WT and KO PML cells to see if the KO of PML caused more DNA damage and there were no significant differences.

Reviewer #3

2. The materials and procedures are generally described only briefly. More detail should be provided in both the Materials and Methods section and in the figure legends to fully appreciate and reproduce the data.

We have addressed this point and our manuscript is now updated to answer this comment.

3. Figure 1 and 2: Can the authors exclude that the partial overlap between some of the IE1 and telomere signals is random rather than reflecting a physical association? How many cells were analyzed for the B panels? Does each dot represent one cell?

- Now figure 2 and 3.
- The number of cells analyzed are indicated in the legends
- Each dot represents a cell
- We exclude that it is not random as we see a significant and reproductible difference between U2OS WT and U2OS KO PML in the colocalization of IE1 at telomeres (Figure 3).

4. IE1-PML binding assays would strengthen the data.

- We have tried Co-IP but could not detect physical interaction. The interaction is either weak or lost during the procedures. However, our BioID data show that PML is in close proximity of IE1B (Figure 1F-G).
- We propose that the interaction between IE1 and PML is not direct but happens through the SUMOylation process as PML is a SUMO E3 ligase which helps bring a SUMO-Ubc9 charged protein to the target protein.

5. Figure 4: Does IE1B affect the number and/or morphology of PML-NBs formed by some but not all PML isoforms? Quantification should be provided (as in Figures 1 and 2).

- Now a supplementary data
- The main objective of this experiment was to determine whether IE1 colocalizes with all VI PML isoforms. We have not observed any effects of IE1 on PML number or morphology.

6. Generation of PML knockout cell lines by CRISPR/Cas9 is critical for the conclusion. However, a number of shortcomings in this approach are present.
   a. The guide RNA is not provided.
   b. It appears that only one gRNA has been used. This is generally less convincing and at least two different gRNAs should be used in separate experiments.
   c. No TIDE analysis is provided.
   d. No control is used. It is stated on p. 18, l. 371-2 that two independent clones were assessed to avoid potential off-target effects. It is stated that these are independent clones. However, no data are provided to demonstrate this. How do the authors know that the two “clones” are not derived from the same cell? Moreover, if these clones are generated with the same gRNA approach using two clones does not address off-target effects. The Off-target effects could be addressed by reconstituting the knockout cell lines with PML.
   f. The CRISPR/Cas9 system may be toxic for the cells. No control with irrelevant gRNA is used to address this aspect.

- The PML KO was done by using plasmids from Santa Cruz biotechnology as described in methods in S4.
- With this assay, two plasmids were provided. One plasmid was for a first guideRNA in the leading strand of exon 1 of PML, the expression of a Cas9n(D10A) and the Puromycin resistance gene. The second plasmid had a guideRNA in the lagging strand overlapping with the first guideRNA, the expression of a Cas9n (D10A) and GFP.
- Both plasmids were transfected in cells and went under puromycin selection for a week. Selected cells were then plate into single cell per well in 96 wells plate to do a single cell cloning assay. Wells were screened every week for the presence of unique colony, if more than two colonies were observed, it was discarded.
- Once single cell clones were amplified, we transferred them into more voluminous wells to amplify the clones and screen for the presence of absence of PML by PCR and sequencing and IFA.
- Hence the reason why no TIDE was provided, it’s because this was already done by the Santa Cruz company.
- These plasmids were also used to generate PML KO cells in another paper^6^.
- For restoration experiments, please refer to comment #4 of reviewer 1.

7. It is not clear what PML is actually doing during integration. Without any mechanisms for how these proteins work, it is difficult to appreciate the potential importance of the present finding.

PML-NBs play a role in repairing DNA double-strand breaks (DSBs) by homologous recombination (HR). Upon infection, the HHV-6B linear viral genomes are likely perceived as broken dsDNA and attempted to be repaired by HR through the action of PML. The presence of telomeric repeats in the viral extremities is the used for repair, leading to viral integration into cellular telomeres. U2OS have many recombination events happening at telomeres, compared to HeLa. Furthermore, it was recently shown that in absence of PML, U2OS have a backup pathway to elongate telomeres through recombination with the help of POLD3 and POLD4 ^2^, explaining, in part, why higher integration frequencies are observed in U2OS relative to HeLa

3. Empty vector control is used for transfection studies, but empty vector is not a sufficient control for effects related to ectopic expression of a protein within the cell.

- We have used another vector (pcDNA4 IE2) as control in some experiments. Furthermore, although they are called empty vector, these do not express the protein of interest but do express other proteins such as Zeocin resistance gene product.

5. It is mentioned that deletion of exon 1 resulted in a short, truncated version of PML (p.14, l.279-280). But it is not demonstrated whether or not this truncated version is actually expressed in the cell, and if expressed whether it can be detected by the antibodies to PML. Without this, it is not clear whether a truncated version may play a role in the PML knockout cells.

- Supplementary figure 4D answers this question.
- Briefly, our antibody detects amino acid 37-51(<https://www.scbt.com/p/pml-antibody-pg-m3>) and the generated truncated protein codes for the first 122 amino acids.

**Minors issuses**

8. Many of the figures could be combined or moved to a supplemental section. The authors might consider merging Figures 1 & 2, Figures 3 & 4, Figures 6 & 8, Figures 9 & 10. Figures 5 and 7 are controls that are more appropriate for supplemental data. The supplemental figures 1 and 2 do not appear to be referenced in the manuscript text.

- We agree. The figures have been merged and rearranged.

9. Text referred to PML interaction with proteins involved in homologous recombination (Lines 117-118) and PML playing a role in DNA repair by recombination (Line 292), but references were not provided until the discussion (Line 340). It is not clear if the literature supports a functional role or just ‘guilt by association’. These roles in DNA repair should be described in the introduction to provide a more clear, logical rationale for these studies.

- Corrections were made.

10. The discussion is lacking an initial summary paragraph of the paper’s findings.

- We added a summary at the beginning of the discussion as suggested.

11. In the section “Material and methods” on “Cell lines and viruses” there is no mentioning of any viruses. Primers and probes for qPCR is not provided.

- Corrected

12. The Author summary has too many technical terms and acronyms to conform to a non-technical summary of the work.

- Corrected

13. Introduction: To my knowledge, viral episome tethering to human chromosomes has only be shown for some gamma-herpesviruses, but not for herpesviruses in general (line 58). The PML-NB components shown to repress viral replication are Daxx, Sp100 and PML (line 106). Rather than de-SUMOylating PML, hCMV IE1 inhibits PML de novo SUMOylation (PMID 27903803).

- Corrected.

14. Results (line 225): The first two sentences are nothing but a platitude (of course permissivity determines whether a virus replicates or becomes latent).
    Corrected
15. Discussion: The authors may want to discuss the possibility that association of IE1 with telomeres may be linked to a potential histone binding activity as shown for hCMV IE1 (PMIDs 24227840, 26812545).

We have performed homology search between the CMV IE1 CRTF and HHV-6B IE1 and found no significant homology. To the extent of our knowledge, IE1 of HHV-6B does not contain a chromatin tethered domain.

16. The main text does not seem to describe and refer to the supporting figures S1 and S2.
    All of our supplementary data are now referred to in the text.

We hope that this revised version will dissipate the majority of concerns initially raised by the reviewers. Considerable work was performed to address the majors concerns and as a result, our manuscript is much improved and provide mechanistic insights associate with HHV-6 integration, a phenomenon that is still largely unknown and poorly characterized.

**References**

1.	Marchesini M, Matocci R, Tasselli L, et al. PML is required for telomere stability in non-neoplastic human cells. *Oncogene*. 2016;35(14):1811-1821. doi:10.1038/onc.2015.246

2.	Zhang J, Yadav T, Zhang J, et al. Alternative Lengthening of Telomeres through Two Distinct Break-Induced Replication Pathways Article Alternative Lengthening of Telomeres through Two Distinct Break-Induced Replication Pathways. *CellReports*. 2019;26(4):955-968.e3. doi:10.1016/j.celrep.2018.12.102

3.	Osterwald S, Deeg KI, Chung I, et al. PML induces compaction, TRF2 depletion and DNA damage signaling at telomeres and promotes their alternative lengthening. *J Cell Sci*. 2015;128(10):1887-1900. doi:10.1242/jcs.148296

4.	Gravel A, Dubuc I, Wallaschek N, et al. Cell Culture Systems To Study Human Herpesvirus 6A/B Chromosomal Integration. *J Virol*. 2017;91(14):e00437-17. doi:10.1128/JVI.00437-17

5.	Reuter N, Schilling E-M, Scherer M, Müller R, Stamminger T. The ND10 Component Promyelocytic Leukemia Protein Acts as an E3 Ligase for SUMOylation of the Major Immediate Early Protein IE1 of Human Cytomegalovirus. *J Virol*. 2017;91(10):e02335-16. doi:10.1128/JVI.02335-16

6.	Milutinovic S, Heynen-Genel S, Chao E, et al. Cardiac glycosides activate the tumor suppressor and viral restriction factor promyelocytic leukemia protein (PML). *PLoS One*. 2016;11(3). doi:10.1371/journal.pone.0152692
