# Supplementary material for: The Promyelocytic Leukemia Protein facilitates human herpesvirus 6B chromosomal integration, immediate-early 1 protein multiSUMOylation and its localization at telomeres
Source: PLoS Pathog. 2020 Jul 13;16(7):e1008683. doi: 10.1371/journal.ppat.1008683 (PMC7394443; doi:10.1371/journal.ppat.1008683)
Supplement: Attachment — Submitted filename: Rebuttal letter_Collin et al 2020_VC_LF.docx [file ppat.1008683.s009.docx]

Dear reviewers,

Thank you for taking the time to review our manuscript and making constructive suggestions. We now provide you a point by point response to the reviewers’ comments and the modifications made to the manuscript.

Part II – Major Issues: Key Experiments Required for Acceptance

**Reviewer #1**

1. The data show that IE1 is hyper-SUMOylated in the presence of PML, but do not show that PML is a SUMO ligase for IE1. Thus, claims like “IE1 is SUMOylated by PML” (lines 23-24) or “PML act[s] as a SUMO ligase for IE1” (lines 28-29) have to be removed from the text or justified experimentally (e.g. by SUMOylation assays with and without PML and/or assays involving an IE1 mutant selectively deficient for PML binding). The positive effect of PML on SUMOylation may be indirect, as recently proposed for hCMV IE1 (PMID 32365141). This is particularly likely, since the interaction between PML and HHV-6B IE1 may be indirect (Figure 1). Perhaps localization of IE1 at PML bodies enhances SUMOylation irrespective of PML acting as a SUMO ligase? In this context, the authors should also acknowledge that their L73E PML mutant may not be selectively deficient for only the SUMO ligase activity but other PML functions as well.

- We thank the reviewer for pointing out the new publication. We agree, and corrections were made by removing those claims and we also changed our title.
- We corrected the overstatements and we suggest that the multiSUMOylation of IE1 is enhanced by the presence of PML-NBs that contains proteins involved in the SUMO pathway.

2. The VIV motif (amino acids 775-777) the authors identified in HHV-6B IE1 may serve an important function, but it is not legitimate to refer to this motif as a SIM (SUMO interacting motif) unless it is shown experimentally that it confers non-covalent interaction with SUMO proteins. Thus, all firm claims that amino acids 775-777 serve as a SIM have to be removed from the manuscript and figures unless the relevant interaction data are provided. In line 215, “773-775” needs to be changed to “775-777”. Instead, it should be clear that the authors are merely speculating that amino acids 775-777 may be part of a SIM. Alternative (or additional) to interacting with SUMO, the VIV motif may be involved in interactions with other proteins. It may, for example, mediate SUMO-independent binding to PML. In hCMV, a similar motif has been implied in interactions with STAT2 and STAT3 (PMID 27387064), and the authors have previously shown that HHV-6B IE1 binds to STAT2 as well (PMID 20404187).

- We agree and corrections were made. Moreover, we suggest that this sequence motif could be a platform for the recruitment of other SUMOylated protein.

3. The data do not show that “IE1 multi-SUMOylation […] is important for IE1 to localize at telomeres” (lines 342-343). A SUMOylation-deficient IE1 mutant needs to be studied to justify this conclusion.

- We agree that this sentence was somewhat misleading and changed it (lines 354-355).
- The use of a SUMOylation-deficient mutant IE1 to assess this is somewhat difficult considering the diffuse pattern of IE1.

**Reviewer #2: (No Response)

Reviewer #3:**

1. An important piece of information is the consequence of PML knock out. The PML knock out cells were apparently tested by PCR (comments to reviewers), please show these data in the supplement figure 5. The cells were, however, tested by IF, but information on the source of anti-PML antibody, including whether it is known to recognize all the isoforms is missing – please provide this information. Moreover, I noticed that in a comment to one of the reviewers it is stated that TIDE was not performed because it was done by Santa Cruz. This appears to be irrelevant, unless Santa Cruz made the cells. A TIDE analysis is performed on the cells after the CRISPR/Cas attempt to knock out the gene, how can Santa Cruz have performed this?

- We’ve added the PCR results in the supplement figure 5F as requested.
- Information about the antibody used is now available in the legend of the supplement figure 5.’’ Sequence in blue represents the length of the protein after deletion of a part of a sequence in exon 1. Truncated protein is from 1-222 amino acids. Sequence in yellow represents the epitope (aa 31-57) recognized by the monoclonal mouse anti-PML antibody used and purchased from Santa Cruz Biotechnology Inc (SC-966)’’
- We agree that TIDE assay could not have been done by Sant Cruz. When responding to the reviewer, we thought that he/she was referring to the Surveyor assay that is typically done when designing guideRNA, which was done by Santa Cruz. We apologize for the confusion and thank the reviewer for pointing this out.

2. Fig. 5B is difficult to understand. What is PML-/- #1 and #2? What does error bar indicate? (used at several places in figure legend without further explanation – please correct) and the y-axis does not give a hint on what has actually been measured (integration). But more troublesome, it is not clear why a Chi-square test has been used and even more difficult to understand that if the error bars indicate the standard deviation, how can a result on three measurements be significant with a p-value of 0.0008 when the error bar for PML-/- #2 encompasses the normalized value for PML+/+. This needs a careful explanation potentially providing raw data.

- PML-/- #1 and #2 refer to clone 1 and clone 2. We changed it to cl.1 and cl.2 to refer as isolated clone 1 and 2 of PML KO cells.
- We agree with the reviewer that it was rather confusing the way we presented the data. Hence, we’ve decided to present the result as tables with actual sample sizes.
- In Table 1 and Table 2, the mean of the percentage of integrated HHV-6B of three independent experiments are shown, representing a total number of cells (n) analyzed for each condition. Total number of HHV-6B integrated cells versus non-integrated HHV-6B cells were compared between condition with a Fisher’s exact test.

Part III – Minor Issues: Editorial and Data Presentation Modifications
**Reviewer #1:**

It would greatly strengthen the manuscript if the authors could get some indication of whether HHV-6B IE1 is important for chromosomal integration.

- We agree and we thank the reviewer for this suggestion. To our knowledge, this would require recombinant HHV-6B (BAC-HHV-6B), which is not yet available. We are working on it though. However, such experiments may be somewhat tricky as we anticipate IE1 to be essential for HHV-6B growth. In that case the use of SUMO-deficient IE1 mutant would be of interest.
- 5. The authors should be more careful in interpreting Figure 5. The average differences between PML +/+ and PML -/- cells in panel B are very small, and the error bars are huge. The results in panel E look more convincing, but why is there such a great difference between the two PML -/- cell lines?
  The large error bars resulted from combining several experiments that were performed over long periods of time. Variations between experiments were important, explaining the large SD. We have now presented the results of a single experiment done in triplicate as tables to facilitate comprehension and minimize error bars. In the results presented, both clones behaved similarly with similar rates of integration.

6. Given the results in Figure 4, it appears inconsistent that “there are no significant changes in the number of IE1 foci between PML +/+ and PML -/- cells” (lines 333-334 and Figure 6E). Please explain.

- We are not certain to understand this comment. In figure 4, we compared WT IE1 with IE1 mutant for nuclear distribution and foci sizes. In figure 6, we analyzed WT IE1 at telomeres in PML+/+ and PML -/- cells. The data indicates that WT IE1 assembles into an equal number of foci whether it is in PML sufficient or PML deficient cells. We do not see an inconsistency in these results.

7. It should be clearer that the mechanisms illustrated in Figure 3B and Figure 4E are hypothetical and that there are alternative interpretations for the presented data. Figure 7 appears to be dispensable. At least, there appears to be major overlap between Figure 3E and Figure 7A, and Figure 7B is somewhat generic.

- We mentioned that these were hypothetical views in the manuscript
- Figure 7 was deleted.

8. Does “IE2A” refer to HHV-6A IE2? Why was an HHV-6A protein used as a control alongside HHV-6B IE1?

- We have added the information in the manuscript (lines 147-149).

9. Provide a reference number for “Osterwalds et al” (line 374). Corrected.

10. The authors have to proof-read their manuscript for spelling mistakes, grammatical errors and other small inconsistencies. There are many of them, even in the Summary (e.g. “Taken together, we could demonstrate that PML act as a SUMO ligase […]”) and Author Summary sections (e.g. “HHV-6B is among few other herpesviruses that integrate its genome in host chromosomes as a mean to establish dormancy”). Other examples include, but are not limited to:
- “what effect it could have on IE1” (line 172);Corrected
- “the presence of PML-I:L73E ” (line 202); Corrected
- “resulting in larger NBs (Figure 4E)” (line 264); Corrected
- “acceptor sites on IE1 (Figure 3D)” (line 241); Corrected
- “phenotypes of IE1 mutants” (line 245); Corrected
- “two distinct cell types” (line 297); Corrected
- “PML deficient cells integrated HHV-6B less frequently” (lines 305-306); Corrected
- “were infected at an MOI of 1” (line 308); Corrected
- “TRF2 may serve to protect” (line 365); Corrected
- “Ubc9 can bind SUMO directly at a SIM” (line 419); Corrected
- “since integration still occurred in PML KO cells, PML’s contribution” (lines 444-445); Corrected
- What does “sewing” mean in line 447? Corrected.

**Reviewer #2: 1. Subject-Verb tense issues.**

a. Line 44: ‘prompt us’ Corrected
b. Line 45: ‘that are essentials’ Corrected

2. Lack of articles.
a. Line 63: ‘Thus far, state’ Corrected
b. Line 83: ‘Furthermore, integrated’ Corrected
c. Line 379: ‘role of PML in DNA repair mechanism Corrected

3. Unclear/poor wording.
a. Lines 82-81: ‘In support, the integrated…’ Corrected
b. Lines 205-207 Corrected
c. Line 223: ‘affected’ is vague, perhaps better described as ‘reduced IE1-SUMO-1 conjugation’ Corrected
d. Lines 262-264: ‘causes’ and ‘causing’ Corrected
e. Lines 341-343: This sentence is misleading- wording tries to walk a fine line that blurs the distinction- be clear that no causal link can be made without the IE1 SUMO mutant. Corrected
f. Lines 408-411: ‘considering’ and ‘considering’ Corrected.
g. Lines 458-461: run-on sentence. Corrected
h. Line 467: ‘auto oligomerization’? Corrected.

4. Use of the possessive for nouns is excessive and not typical for science communications.
Examples are line 113, 170, 245,408,1070,1079, 1081, 1104, 1179, 1180 but found throughout manuscript. Corrected.

5. Lines 122-124- put citation directly after author’s name ‘Stanton et al.’ Corrected.

6. Line 145 correct to ‘we studied the’ Corrected.

7. Typos
a. Line 156 ‘pull downed’ Corrected.
b. Line 157 ‘my mass spectrometry’ Corrected.
c. Line 167 ‘Taking together’ Corrected.
d. Line 184 ‘mono, poly or. ‘Corrected.
e. Be consistent with using ‘Figure’ or ‘figure’ Corrected.
g. Line 376 ‘We’ve’- conjugations should be avoided Corrected.
h. Line 475 ‘lost time’ Corrected.
i. Line 1079 ‘SUMOylation is enhance’ Corrected.
j. Line 1097, 1140, 1145 and elsewhere ‘means’ Corrected.
k. Line 1114 ‘and processes’ Corrected.

m. Line 1143 ‘A qPCR was done’ Corrected

8. SUMO 1, 2, 3 not detailed in Figure 2B. Corrected

9. Bar graph for Figure 2F is a bit oversized and overkill since there is only a low amount of signal for the one condition. The graph was deleted.

10. Break in paragraph at line 215 not needed. Corrected

11. Lines 267-268, specify in U20S cells Corrected

12. The rationale for figure 6 is confusing. Clearly state U20S cell are ALT+ at beginning of paragraph. Corrected

Reviewer #3:

I cannot read from the methods how random (unbiased) selection of nuclei was performed for the confocal data? Given the great variability (also seen in fig. 1B IE foci) how can conclusions of number of foci in time be achieved when only to measurements is performed? (again fig. 1B IE foci at 24 hrs).
Therefore, the statement l.129 “the number of foci diminishes with time” appears not to be supported by the data for IE foci and should be removed, or more data points should be included. We removed the figure as it was not our primary message.

l.30: …by these viruses to maintain their… -> …by this virus to main its…? Corrected
l.66: HHV6-B -> HHV-6B Corrected
l.86: characterizes -> characterized Corrected
l.88: ref 18 is missing volume and page numbers. Corrected
l.142: “show perfect colocalization”, and in l. 144 about the same finding: “almost perfect correlation”? Both cannot be true. Please characterize the colocalization in a more scientific manner. Corrected
l.184: remove the period after “or.” Corrected
